# The effect of Bosentan on exercise capacity in Fontan patients; rationale and design for the TEMPO study

**DOI:** 10.1186/1471-2261-13-36

**Published:** 2013-05-11

**Authors:** Anders Hebert, Annette S Jensen, Lars Idorn, Keld E Sørensen, Lars Søndergaard

**Affiliations:** 1 Department of Cardiology, dep. 2014, Copenhagen University Hospital, Rigshospitalet, Blegdamsvej 9, 2100, Copenhagen, Denmark; 2 Department of Cardiology, Aarhus University Hospital, Skejby, Brendstrupgaardsvej 100, 8200, Aarhus N, Denmark

**Keywords:** Univentricular heart, Fontan, Exercise capacity, Endothelin receptor antagonist, Randomized placebo-controlled trial

## Abstract

**Background:**

Palliative treatment with the Fontan procedure has greatly improved survival for children with functionally univentricular heart. Since Fontan performed the first successful operation, the procedure has evolved and is now performed as Total Cavo-Pulmonary Connection (TCPC).

An increasing prevalence and longer life expectancy of TCPC patients have raised new challenges. The survivors are often suffering complications such as arrhythmias, myocardial dysfunction, thromboembolic events, neuropsychological deficit, protein-losing enteropathy and reduced exercise capacity. Several causes for the reduced exercise capacity may be present e.g. impaired function of the single ventricle, valve dysfunction and chronotropic impairment, and perhaps also increased pulmonary vascular resistance. Thus, plasma endothelin-1 has been shown to correlate with increased pulmonary vascular resistance and the risk of failing Fontan circulation. This has raised the question of the role for pulmonary vasodilation therapy, especially endothelin receptor antagonist in the management of TCPC patients.

**Methods/Design:**

The TEMPO trial aims to investigate whether Bosentan, an endothelin receptor antagonist, can be administered safely and improve exercise capacity in TCPC patients. The trial design is randomized, double-blind and placebo-controlled. Bosentan/placebo is administered for 14 weeks with control visits every four weeks. The primary endpoint is change in maximal oxygen consumption as assessed on bicycle ergometer test. Secondary endpoints include changes in pulmonary blood flow during exercise test, pro brain natriuretic peptide and quality of life.

**Discussion:**

We hypothesize that treatment with Bosentan, an endothelin receptor antagonist, can be administered safely and improve exercise capacity in TCPC patients.

**Trial registration:**

clinicaltrials.gov NCT01292551

## Background

Functionally univentricular hearts (UVH) include congenital defects with hypoplasia of either the right or the left ventricle, which, as a consequence, cannot drive a pulmonary or systemic circulation alone. The natural history of UVH is poor with reported one-year survival rates less than 50% [1]. Introduction of palliative surgical treatment by Fontan in 1971 [2] has dramatically improved survival. The technique has evolved [3,4] and is currently performed as Total Cavo-Pulmonary Connection (TCPC). Although the procedure remains palliative rather than curative, the 10-year survival rate post-surgery increased from 50% in the first Fontan patients to 90% currently [5-7].

The complete TCPC is typically carried out in three stages. Generally, the first stage is undertaken in the neonatal period and aims at regulating the pulmonary blood flow to an adequate level, either by reducing or increasing pulmonary flow using a pulmonary banding or a shunt, respectively, but also securing that systemic perfusion and mixing remains unobstructed. The second stage is the bidirectional Glenn procedure, where the superior vena cava is anastomosed to the pulmonary artery. In the third stage, the TCPC circulation is established by diverting the inferior vena cava to the pulmonary artery, thus allowing almost complete systemic venous bypass of the heart. The second and third stages are usually performed between six months and three years of age.

The prevalence of TCPC patients has steadily increased over the past decades. A recent Danish population-based study found 43 surviving UVH patients per million inhabitants, 37 of whom were palliated with Fontan/TCPC [8]. Despite improved survival, TCPC is still associated with high morbidity. Myocardial dysfunction, arrhythmias, thromboembolic events, neuropsychological deficits, protein-losing enteropathy, and reduced exercise capacity are among the most commonly reported long-term complications [9-12].

In the TCPC, pulmonary flow is driven without support from a sub-pulmonary ventricle. Thus, even a slight increase in pulmonary vascular resistance (PVR) may compromise the pulmonary circulation which in turn may result in decreased preload of the ventricle, limiting cardiac output (CO) and exercise tolerance [13,14]. An increased pulmonary blood flow prior the Glenn anastomosis may contribute the elevated PVR in some patients. Furthermore, the non-pulsatile pulmonary blood-flow following the TCPC procedure may also increase PVR through remodeling, as shown in an animal model [15]. This is supported by the fact that raised levels of the potent vasoconstrictor endothelin-1 correlate with increased PVR and failure of the Fontan circulation [11,16]. Furthermore, endothelin-1 is elevated in many UVH patients before surgical palliation [17]. This raises the question whether pulmonary vasodilators may have a role in the treatment of this patient group.

Endothelin receptor antagonists (ERA) not only have an immediate vasodilating effect on the pulmonary vasculature, but also a long term effect in its inhibition of the structural remodeling of the pulmonary microcirculation, thus theoretically improving exercise capacity in patients with TCPC circulation. Accordingly, small studies have shown that oral treatment with pulmonary vasodilators, including ERAs improves hemodynamics and exercise capacity after TCPC operation [18-21].

The TEMPO trial is a randomized, placebo-controlled and double-blinded study. The aim is to investigate whether Bosentan, an ERA, can improve exercise capacity and is safe to administer to TCPC patients.

## Methods/Design

### Participants

The participants are recruited among all registered TCPC patients over 12 years of age from the four participating tertiary centers; two Danish centers; Copenhagen University Hospital, Rigshospitalet and Aarhus University Hospital, Skejby and two Swedish centers; Karolinska Institutet in Stockholm and Lund University Hospital. Subjects who fulfill the inclusion criteria and do not meet any exclusion criteria (Table 1) are contacted, aiming for 78 participants. All participants receive written and oral information in their own language, and sign an informed consent form before enrollment.

**Table 1:** **In**-/**exclusion criteria**

**Inclusion criteria**	
•	Fontan/TCPC operated
•	Expected clinical stability > 3 months, evaluated by investigator from clinical record
**Exclusion criteria**	
•	Age < 12 years
•	Severe heart failure (NYHA-class IV)
•	Oxygen saturation < 85% at rest
•	Pre-existing liver condition (transaminases 2x > upper reference limit)
•	Renal failure (creatinin > 150 mmol/l)
•	Restrictive Fontan circuit
•	History of work induced severe arrhythmia
•	Systolic blood pressure below 80% of reference (< 88 mmHg)
•	Use of any of following drugs: Fluconazole, Ketoconazole, CyclosporineA, Lopinavir, Ritonavir, Rifampicin, Carbamazepine and Phenytoin
•	Severe extra-cardiac condition e.g. neurological impairment
•	For women: Positive HCG or no use of contraception*
•	History of low compliance

TCPC: Total Cavo-Pulmonary Connection, NYHA: New York Heart Association, HCG: Human Chorionic Gonadotropin, *: contraception includes celibacy.

Demographic and diagnostic data as well as blood samples are recorded at baseline (Table 2).

**Table 2:** Baseline data

**Demographic and diagnostic**	
Gender	Male/female
Age	Years
Ventricular anatomy	Right/left/mixed
Basic diagnosis	
Patent fenestration	Yes/no
Current medication	
**Blood sample**	**Stool sample**
Liver enzymes	Fecal α1-antitrypsin
Albumin	
Protein	
Pro-Brain Natriuretic Peptide	
C-terminal proendothelin-1	

### Endpoints

Endpoints are given in Table 3. Maximal oxygen consumption (peakVO_2_), pulmonary blood flow, oxygen consumption at anaerobic threshold (VO_2_ [AT]) and maximal work load are all calculated from results of a bicycle ergometer test, performed at Institute of Sports Medicine Copenhagen, as described below. Blood samples and SF36 quality of life questionnaire are obtained at the baseline and endpoint visits. NYHA class is determined before the bicycle test. Fecal samples for α-1 antitrypsin are collected by the participant at home and subsequently sent in a provided envelope for analysis. It is optional for participants to partake in this endpoint.

**Table 3:** Endpoints

**Primary endpoint**	
•	peakVO_2_ (ml/min, ml/min/kg body weight)
**Secondary endpoints**	
•	VO_2_ (AT) (ml/min)
•	Maximal load (Watt)
•	Pulmonary blood flow (by Stringer-Wassermann method)
•	NYHA class
•	SF36 quality of life questionnaire
•	proBNP (pmol/l)
•	Fecal α1-antitrypsin (g/kg)

PeakVO2: maximal oxygen consumption in test, VO_2_ (AT): oxygen consumption at anaerobic threshold, NYHA: New York Heart Association, proBNP: pro-Brain Natriuretic Peptide.

### Bicycle ergometer test

The primary endpoint is improvement in peakVO_2_ (ml/min and ml/min/kg body weight) obtained during a bicycle ergometer test. All tests are performed on the same equipment (Monark Ergomedic 839E, Monark Exercise AB, Sweden), at the same site and with the same staff present. An individualized incremental protocol is chosen for each patient, with the purpose of reaching maximal effort within 6–12 minutes. All participants start with one to two minutes warm-up. After this, the test protocol starts at either 20 Watt increasing stepwise 20 Watt/min or starting at 25 Watt increasing 25 Watt/min. Before each test the flow-meter and gas analyzer are calibrated. During the test electrocardiography and percutaneous oxygen saturation are measured continuously. The participants wear a mask with a TripleV flow-meter (Erich Jaeger GmbH, Germany) to measure respiration volume, and a tube for breath-by-breath sampling to measure O_2_ and CO_2_ concentrations via Masterscreen CPX (CareFusion, San Diego, CA, USA). All measures are given as the average over 15 seconds intervals subtracting the highest and lowest value. Pulmonary blood flow is calculated continuously by the test software using the Stringer-Wassermann method [22]. Only participants without patent fenestration of the TCPC tunnel are considered for this endpoint.

The principal investigator is present at all tests to motivate patients to reach maximal effort in both pre- and post-treatment tests. Furthermore, Respiratory Exchange Ratio (RER) is measured, and a value of ≥1.1 is considered acceptable as an indicator of maximal effort. Patients who are unable to reach RER ≥1.1 in either the pre- or post-treatment test will not be considered in the statistical analysis for the primary endpoint. PeakVO_2_ is defined as the 15 second interval with the highest value of VO_2_.

Anaerobic threshold is defined as the first 15 second interval where the average RER passes the value 1.0 without falling below 1.0 later [23].

It is considered unnecessary to make the participants undergo a “dummy test” before enrollment since patients in the Scandinavian countries are familiar with bicycles, and exercise testing is frequently used in the clinical follow-up of TCPC patients. Furthermore, this was confirmed in a recent feasibility study where six Danish patients with congenital heart disease and one healthy adolescent were tested twice within six weeks, demonstrating excellent reproducibility, with an intra-class correlation coefficient of 0.99 [24].

### Trial design

**Figure 1:**
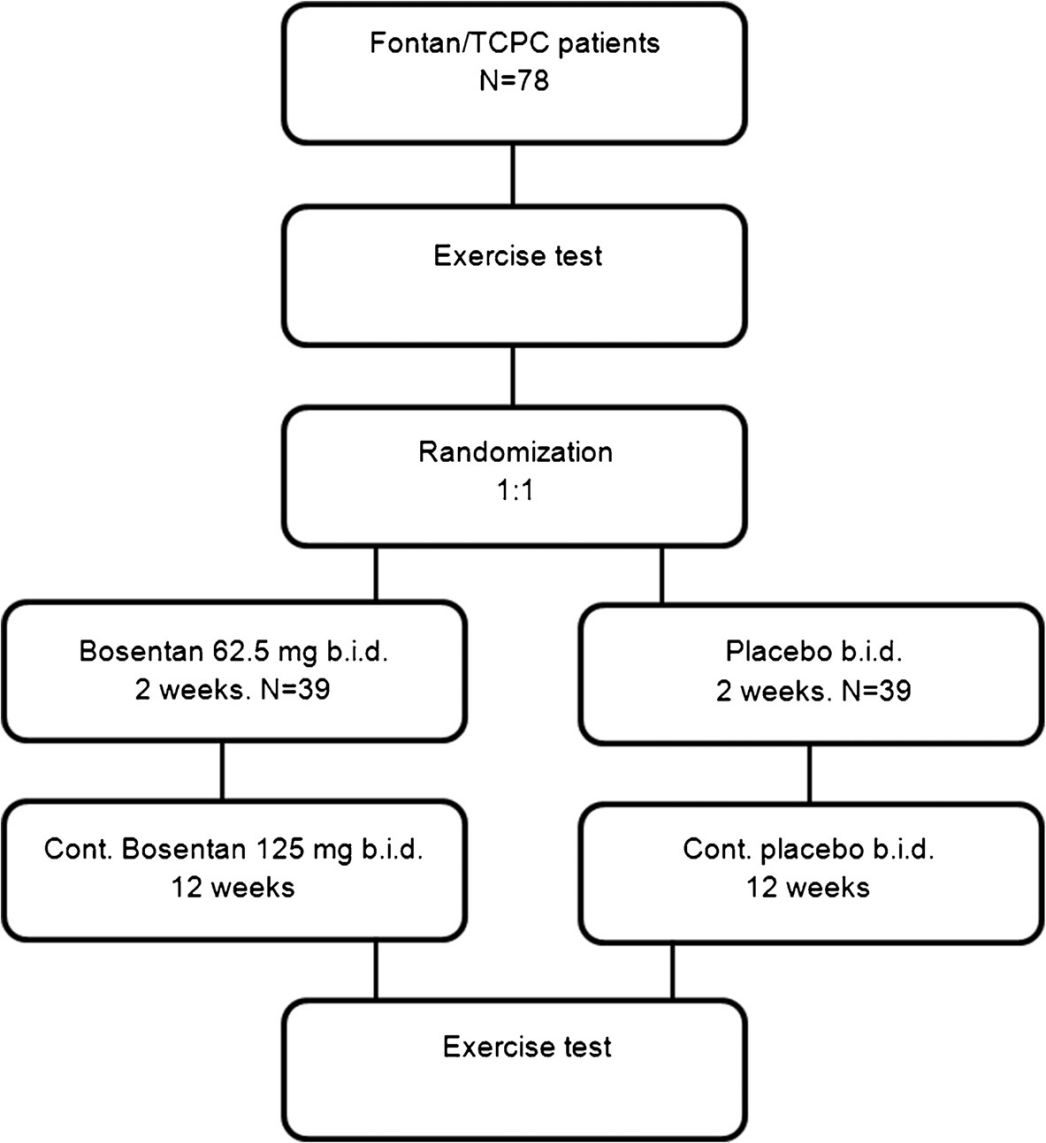
**Study design. **b.i.d.: twice daily, Cont.: continue.

The trial is a randomized, double-blinded, placebo-controlled clinical trial. Patients with TCPC palliated UVH, are eligible for enrollment. Participants are randomized 1:1 to parallel groups of treatment or placebo (Figure 1). The Bosentan dose regimen is the same as used in patients with pulmonary arterial hypertension [25]. Participants receive oral placebo or Bosentan 62.5 mg b.i.d. for two weeks followed by 125 mg b.i.d. for 12 weeks. For patients with a body weight below 35 kg, the dose is halved i.e. 31.25 mg b.i.d. for two weeks and 62.5 mg b.i.d. for 12 weeks.

### Medication/Randomization

At enrollment participants receive a package with four glasses each containing 70 tablets; one with placebo or Bosentan 62.5 mg tablets for the first two weeks, and three with placebo or Bosentan 125 mg for the remaining 12 weeks. The study medication is delivered by Actelion Pharmaceuticals packed, labeled and numbered. The packages are randomized when received by the investigators. Randomization is done via a computerized model, and only the manufacturer knows the randomization status.

At each visit the remaining tablets are counted to control compliance. Patients who take at least 80% of the intended tablets are defined as “per protocol” whereas the rest are counted as “intention to treat”.

### Safety/visits

During the study, participants will have a total of five visits (Table 4), three of which are intermediary controls to ensure safety. All tests at the baseline and final visit are performed in Copenhagen, whereas the first intermediary visit after two weeks must take place in one of the four participating tertiary centers. Control visits may take place at a local hospital or at the general practitioner’s office, in case this is more convenient to the participant.

**Table 4:** Visits

**Visit/week**	**Baseline**	**Increase medication**	**Safety visit**	**Safety visit**	**Endpoint**
Place	Cph	Tertiary	Local	Local	Cph
Time window	Day 0	Day 10-18	Day 35-49	Day 63-77	Day 91-105
Bosentan dose after visit	62.5 mg x 2	125 mg x 2			None
In-/exclusion criteria, informed consent	X				
Exercise test, SF36 q.o.l., α1-AT	X				X
Blood pressure, heart rate, SAT	X	X	X	X	X
Blood pressure/hypotension control	X	X			
Compliance control and adverse effects		X	X	X	X
CT-proET-1, IgA, IgG, Pro-BNP, albumin, protein, LDH	X				X
ALAT, ASAT, bilirubin, creatinine, PCV, Hb, thrombocytes, (women) HCG	X	X	X	X	X

Cph: Copenhagen University Hospital, Rigshospitalet, Tertiary: Any of the four participating tertiary centers, Local: Local hospital including tertiary center or general practitioners office, SF36 q.o.l.: SF36 quality of life questionnaire, α1-AT: fecal α1-antitrypsin, SAT: transcutaneous oxygen saturation, CT-proET-1: C-terminal proendothelin-1, IgA-G: Immunoglobulin A-G, Pro-BNP: Pro-Brain Natriuretic Peptide, LDH: Lactate dehydrogenase, ALAT/ASAT: Alanine-/aspartate aminotransferase, PCV: Packed cell volume/hematocrit, Hb: Hemoglobin, HCG: Human chorionic gonadotropin.

When starting medication at baseline and when increasing the dose after two weeks a special safety measure is taken. Each participant has blood pressure, heart rate and percutaneous oxygen saturation measured before the medication is given. Blood pressure is then measured one and two hours after the medication is administered. If symptoms of hypotension and a decrease in systolic blood pressure over 20% are seen, the participant will be evaluated by clinical experts and possibly excluded.

Female participants are advised about contraception methods, and encouraged to contact the study group with any suspicion of pregnancy. At every visit s-hCG is measured.

In case of serious adverse events the medication is stopped and the patient is excluded. Moreover, adverse events (Table 5) lead to exclusion of the study or continuation at a lower dose of medication.

**Table 5:** Safety measures

**Observation**	**Action**
Systolic blood pressure < 80 mmHg	Exclusion
Hemoglobin < 6.5 mmol/l	Exclusion
> 10% decrease in oxygen saturation	Exclusion
> 10% decrease in packed cell volume	Exclusion
> 10% decrease in thrombocytes	Exclusion
Two times increase in bilirubin*	Exclusion
Liver transaminases 3–5 times reference limit	Continue in study at half dose of medication
Liver transaminases >5 times reference limit	Exclusion

*In patients with bilirubin at baseline within the reference limits an increase to 2 x upper reference limit leads to exclusion; if bilirubin is already over reference limit at baseline, a bilirubin increase to 2 x the baseline value leads to exclusion.

### Sample size calculation

Average peakVO_2 _in adult TCPC patients is approximately 20 ml/kg/min [10]. Assuming that 14 weeks treatment with Bosentan compared to placebo will increase in the primary endpoint peakVO_2 _by 20%, an increase of 4 ml/kg/min is expected.

The standard deviation (SD) of a single measurement of maximal VO_2 _in TCPC adults is reported to range from 2.5 to 5.8 ml/kg/min [10,18]. Thus, a weighted mean SD of 4.3 ml/kg/min is used in the calculations in this study.

The intra-patient correlation is assumed to be 0.5, and the required power is 90% at a significance level of 95%. With a paired *t*-test, the number needed in each group is 24. Exclusion due to elevated liver enzymes is expected in 5%, low medication compliance in 15%, whereas drop-outs are expected in 33% of the participants. Thus, 39 patients will be included in each group.

## Discussion

Surgical and medical advances in recent decades have improved the survival of children born with UVH; hence, we can expect a higher prevalence and an increasing age of patients palliated with TCPC in the future. When older, impaired exercise capacity may be an important long-term complication in TCPC patients. The TEMPO study hypothesizes that the ERA Bosentan partly may counteract this effect, particularly considering the finding of high plasma levels of endothelin-1 in UVH and TCPC palliated patients.

Current evidence is pointing toward the lack of preload reserve as the most important factor in limiting CO in TCPC patients [13,14]. In the healthy biventricular individual, the CO is readily increased with a reduction in afterload, augmentation of ventricular contractility or increase in heart rate. The effect of these factors is dependent on an abundant preload reserve, in the absence of which CO increase would suffer. In the Fontan circulation there is limited preload reserve due to the passive pulmonary flow. In a ramp exercise test, an increase in heart rate will reflect positively on CO as long as the preload is kept intact. Since this may not be the fact in a TCPC circulation, any further increase in heart rate will more likely decrease the stroke volume. The lowering of PVR may increase pulmonary blood flow and thereby preload reserve in Fontan patients, and due to this pulmonary vasodilating therapy is interesting as a means to improve the Fontan circulation. The limited preload reserve is most likely to stem from PVR in the “healthy Fontans”, but in cases where the Fontan conduit itself is restrictive, lowering the PVR would not be effective. As a consequence, patients with documented restrictive Fontan circuit on prior imaging are excluded from this study.

Pulmonary vasodilation therapy in the form of Sildenafil was previously evaluated in a placebo-controlled trial with TCPC patients both by Giardini et al. [18] and Goldberg et al. [26] showing an increase in peakVO_2_ and ventilatory efficiency, respectively. An open-label study on the effect of ERA in TCPC patients showed no effect on peakVO_2_[27]; however, the study population was relatively small with only 32 patients finishing with endpoint. Furthermore, the safety of ERA treatment to TCPC patients has recently been evaluated in an open-label pilot study [28]. Thus, the TEMPO-study is the first randomized, placebo-controlled study to evaluate the safety and effect of ERA on peakVO_2 _in a TCPC population.

The primary endpoint of this trial is peakVO_2_; a test that is notoriously difficult to reproduce reliably, partly due to the fact that tests are often performed on different equipment, with different staff present, and with different levels of motivation. The quality of the primary endpoint data is of utmost importance, and testing all patients at the same location, with the same equipment and staff should secure the highest level of inter-test reliability.

In order to get a better understanding of the effect or lack of effect of ERA on the Fontan circulation, this study uses the non-invasive estimate of CO during exercise proposed by Wassermann et al. [22]. Though proven very reliable in healthy individuals as well as in patients with chronic heart failure [29,30], this method has to the investigators’ knowledge not yet been validated in Fontan patients. However, considering the nature of the method it is quite sane to assume that it will be as accurate and precise in Fontan patients as it is in chronic heart failure and healthy individuals.

The method is based on the fact that the difference in blood O_2_ content from arterial to mixed venous (C[a-vDO_2_]) develops linearly in relation to change in oxygen consumption (VO_2_) from rest (0%) to maximum uptake (100%) by the following regression line:

$$C\left(a-vD_{O_{2}}\right)=5.72+0.105*\%{\dot{V}}_{O_{2\max}};\cdot r=0.94$$

VO_2_ is measured continuously, so CO can be calculated using the direct Fick method:

$$\mathrm{CO}=\frac{{\dot{V}O}_{2}}{C\left({\mathrm{a-vD}}_{O_{2}}\right)}$$

In Fontan patients without significant shunts, virtually all the blood passes through the pulmonary circulation, as is the case in a biventricular circulation without shunts. None of the assumptions of the method are violated in the Fontan circulation without shunts.

### Ethical considerations

The Danish Ethics Committee, the Danish Medicines Agency, and the Danish Board for Data Protection approve the study. The Swedish Ethics Committee are informed about the study, and agree with the Danish Committee without making a separate approval, as all the endpoint tests are done in Denmark and the medicine is delivered to the patients in Denmark. This means that the study takes place in Denmark only, but with Swedish patients participating under the Danish laws of patient rights and under Danish insurance.

### Monitoring

The study complies with the standards of Good Clinical Practice ICH-GCP. The GCP unit of Copenhagen monitors all aspects of the study according to the guidelines of ICH-GCP including initiation visit, planned intermediary control visits and end-of-study visit. Furthermore they can make visits unannounced if need be.

## Abbreviations

UVH: Univentricular heart; TCPC: Total cavo-pulmonary connection; PVR: Pulmonary vascular resistance; CO: Cardiac output; ERA: Endothelin receptor antagonist; VO2: Oxygen consumption; AT: Anaerobic threshold; NYHA: New York Heart Association; RER: Respiratory exchange ratio; b.i.d.: Bis in die (twice daily); s-hCG: Serum human chorionic gonadotropin; SD: Standard deviation.

## Competing interests

Actelion Pharmaceuticals has produced and provided the study medication and also supports the trial with an unrestricted grant. None of the authors have any interest in Actelion Pharmaceuticals. Actelion Pharmaceuticals neither has any ownership over the obtained data, nor any involvement in data processing or writing of the final report.

## Authors’ contribution

AH: Wrote the protocol, contacted eligible participants, conducted interviews and tests of all participants at baseline and endpoint and did the statistical analyses. Also wrote the manuscript. ASJ: Helped writing the protocol and articles and has provided academic support. LI: Helped by providing contact information and baseline characteristics in all eligible patients from Denmark and has provided academic support. KS: Helped recruiting participants and conducted the intermediary visits in all participants from West Denmark. LS: Conceived of the study, and participated in its design and coordination and helped to draft the protocol and manuscript. All authors read and approved the final manuscript.

## Pre-publication history

The pre-publication history for this paper can be accessed here:

http://www.biomedcentral.com/1471-2261/13/36/prepub
